# Association between the *TGF-β1* polymorphisms and chronic obstructive pulmonary disease: a meta-analysis

**DOI:** 10.1042/BSR20170747

**Published:** 2017-08-31

**Authors:** Ning Liao, Hua Zhao, Min-Li Chen, Zheng-Fu Xie

**Affiliations:** Department of Geriatrics and Gerontology, First Affiliated Hospital, Guangxi Medical University, Nanning, China

**Keywords:** Chronic obstructive pulmonary disease, meta-analysis, polymorphism, transforming growth factor-β1

## Abstract

It has been hypothesized that polymorphisms in the transforming growth factor-β1 (*TGF-β1*) gene on chromosome 19 modify the risk for chronic obstructive pulmonary disease (COPD). However, results from previous studies are contradictory. We therefore conducted a meta-analysis of published case–control studies on the association between five common *TGF-β1* polymorphisms (rs1982073, rs1800469, rs2241712, rs6957, and rs2241718) and COPD risk. Data sources were Pubmed, Scopus, ISI Web of Science, China National Knowledge Infrastructure (CNKI), and Wanfang databases. Twelve studies including 6749 participants were reviewed and analyzed. For the *TGF-β1* polymorphism rs1982073, the results indicted that the C allele was associated with decreased risk of COPD in Caucasians (odds ratio (OR) =0.79, 95% confidence interval (CI): 0.64–0.99, *P*=0.038) but not in Asians (OR =0.95, 95% CI: 0.71–1.28, *P*=0.741). No associations with COPD were identified for other polymorphisms evaluated in the present study including rs1800469 (T allele compared with C allele, OR =0.89, 95% CI: 0.77–1.02, *P*=0.099), rs2241712 (A allele compared with G allele, OR =1.03, 95% CI: 0.89–1.20, *P*=0.666), rs6957 (A allele compared with G allele, OR =1.14, 95% CI: 0.95–1.36, *P*=0.160), and rs2241718 (C allele compared with T allele, OR =0.95, 95% CI: 0.79–1.14, *P*=0.571). In conclusion, this meta-analysis showed that the C allele of rs1982073 was protective against COPD in Caucasians but not in Asians, whereas there was no association of rs1800469, rs2241712, rs6957, and rs2241718 with COPD.

## Background

Chronic obstructive pulmonary disease (COPD) is characterized by persistent progressive airflow limitation, chronic inflammation of the airways, and systemic effects or comorbidities. Genetic factors play a role in COPD susceptibility but are poorly understood [1]. Transforming growth factor-β1 (TGF-β1) is a pleiotropic cytokine that regulates a wide range of biological functions including proliferation and differentiation of cells, extracellular matrix formation, gene transcription, protein biosynthesis, and tissue repair [2]. In addition, TGF-β1 is an important regulator of inflammatory responses and immunological homeostasis. Experimental studies have shown that TGF-β1 in the respiratory mucosa can profoundly reduce inflammation and airway hyperreactivity [3]. In patients with COPD, TGF-β1 expression was increased in bronchiolar and alveolar epithelium, and the epithelial expression of TGF-β1 correlated with the number of intraepithelial macrophages and mast cells [4]. In addition, the levels of TGF-β1 were positively correlated with the extent of COPD patients’ smoking history and the degrees of peripheral airway obstruction [5]. Therefore, TGF-β1 may be involved in the pathogenesis of COPD. TGF-β1 is encoded by the *TGF-β1* gene located in chromosome 19q13.1-q13.3. A number of studies have assessed the relation of single nucleotide polymorphisms (SNPs) in the *TGF-β1* gene with COPD; however, results have been controversial and the contribution of these SNPs to COPD risk remains unclear [6–17]. To clarify this matter, we analyzed data from published case–control studies using a meta-analysis study design.

## Materials and methods

### Literature search

We adopted the Preferred Reporting Items for Systematic Reviews and Meta-analysis (PRISMA) guidelines for our meta-analysis. Literature searches were conducted to identify relevant studies in the following sources: PubMed, Scopus, ISI Web of Science, China National Knowledge Infrastructure (CNKI), and Wanfang databases until 25 April 2017. The following terms were used in the search string: ‘chronic obstructive pulmonary disease’, ‘COPD’, ‘transforming growth factor-β1’, ‘TGF-β1’, ‘polymorphism’, ‘genetics’, and ‘association study’. The literature was screened and selected for eligibility and inclusion on the basis of title, abstract and keywords by two independent reviewers. Disagreements were resolved by consensus. When a title/abstract could not provide us with enough information for study selection, the full text of the publication was evaluated. To further identify potentially relevant studies missed by the electronic database search, the bibliographies of relevant meta-analyses and reviews were hand searched. When two similar studies were reported from the same institution or author, the study with the largest dataset was included in the meta-analysis. We did not apply for ethics approval in that we only utilized previously published data.

### Inclusion criteria

Our meta-analysis included genetic association studies fulfilling the following inclusion criteria: (i) study design: case–control studies, (ii) investigating the association of the *TGF-β1* polymorphisms with COPD, (iii) providing information on genotypic or allelic frequency for the *TGF-β1* polymorphisms in both COPD patients and control subjects, and (iv) genotype distribution in the control group was in agreement with Hardy–Weinberg equilibrium (HWE) expectations. Criteria for exclusion were as follows: case reports, editorials, review articles, meeting abstracts, familial-based studies, and animal studies.

### Data extraction and quality assessment

Two reviewers were involved in data extraction in a predetermined database. The following data were extracted from each study: first author, year of publication, country, ethnicity, and genotypic or allelic distribution of the *TGF-β1* polymorphisms in cases and controls. Disagreement between the reviewers was resolved by discussion. The Newcastle–Ottawa Scale (NOS) was used to assess the quality of each study [18]. Included studies were judged on eight items across three key areas: selection of cases and controls, comparability of the participants, and outcomes. Ratings were summed up to a total score with a maximum value of 9 points, and a score of ≤5 indicated high risk of bias [19].

### Analysis of data

Quantitative meta-analysis was performed using STATA version 11.0. The odds ratio (OR) was used as a summary statistic. We calculated four crude unadjusted ORs and their corresponding 95% confidence intervals (CIs) for each study: (i) dominant OR, (ii) recessive OR, (iii) homozygote OR, and (iv) allele contrast OR. A DerSimonian and Laird random effects meta-analysis model was used to estimate the pooled effect sizes in case of substantial between-study heterogeneity [20]. In this manner, inverse of the variance of each study was used as the weight for the study. The *Z*-test was used to calculate the *P*-value of the overall effect for the meta-analysis and *P*<0.05 was considered statistically significant. Included studies were tested for between-study heterogeneity using the chi-square test, with significance set at *P*<0.10 [21]. We excluded each study in turn and reran meta-analyses to investigate the impact of each study on the combined effect. In order to test for publication bias, we utilized the Begg’s test. We did not perform funnel plots to evaluate publication bias because there were fewer than ten studies that qualified for each SNP [22].

## Results

### Study selection and characteristics

**Figure 1:**
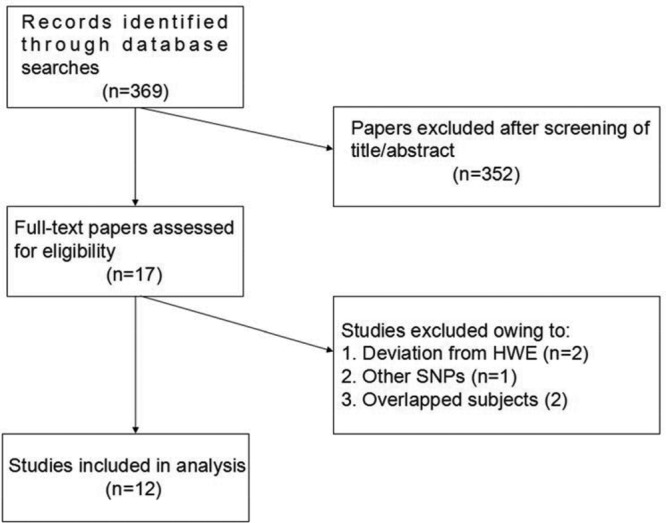
Flow diagram of the screening process of retrieved studies

In the present meta-analysis, we aimed to evaluate the relation of five common SNPs (rs1982073, rs1800469, rs2241712, rs6957, and rs2241718) in the *TGF-β1* gene with COPD. Three hundred and sixty-nine (*n* =369) unique papers were retrieved from searching five major databases (Figure 1). After screening for relevance based on title and/or abstract, 17 papers were identified as potentially eligible and underwent a full text review. Five studies were excluded in the full-text screening for not having met the inclusion criteria: two studies included overlapped participants [23,24], two studies contained control subjects showing evidence of departure from HWE [25,26], and one study assessed other SNPs [27]. Finally, 12 studies met the inclusion criteria and were included in the quantitative analysis. These studies were conducted between 2005 and 2012. The combined population size of the 12 studies totalled 6749 individuals (COPD patients: *n*=2952; controls: *n*=3797). Study characteristics and methodological quality of included studies are presented in Table 1.1, 1.2, 1.3, 1.4, and 1.5.

**Table 1.1:** Characteristics of the studies of rs1982073 and COPD risk

Author	Country	Year	Ethnicity	Cases (*n*)	Controls (*n*)	Control origin	Cases					Controls					Score*
							TT	TC	CC	T allele (%)	C allele (%)	TT	TC	CC	T allele (%)	C allele (%)	
Wu	New Zealand	2004	Caucasian	165	140	Hospital based	69	83	13	221 (67)	109 (33)	41	73	26	155 (55)	125 (45)	7
Celedon	U.S.A.	2004	Caucasian	304	441	Population based	NA	NA	NA	389 (64)	219 (36)	NA	NA	NA	494 (56)	388 (44)	8
van Diemen	Netherlands	2006	Caucasian	170	1071	Population based	75	72	23	222 (65)	118 (35)	382	533	156	1297 (61)	845 (39)	7
Yoon	Korea	2006	Asian	102	159	Hospital based	21	61	20	103 (50)	101 (50)	33	75	51	141 (44)	177 (56)	8
Ito	Japan	2008	Asian	70	99	Hospital based	NA	NA	NA	78 (56)	62 (44)	NA	NA	NA	105 (53)	93 (47)	7
Mak	China	2009	Asian	201	195	Population based	38	92	71	168 (42)	234 (58)	39	93	63	171 (44)	219 (56)	7
Liu	China	2010	Asian	219	148	Not mentioned	60	105	54	225 (51)	213 (49)	50	81	17	181 (61)	115 (39)	7
Chappell	U.K.	2011	Caucasian	1017	912	Not mentioned	NA	NA	NA	1220 (60)	814 (40)	NA	NA	NA	1094 (60)	730 (40)	7
Yuan	China	2011	Asian	117	82	Hospital based	31	61	25	123 (53)	111 (47)	10	47	25	67 (41)	97 (59)	7

* Score of NOS. Abbreviation: NA, not applicable.

**Table 1.2:** Characteristics of the studies of rs1800469 and COPD risk

Author	Country	Year	Ethnicity	Cases (*n*)	Controls (*n*)	Control origin	Cases					Controls					Score*
							CC	CT	TT	C allele (%)	T allele (%)	CC	CT	TT	C allele (%)	T allele (%)	
Celedon	U.S.A.	2004	Caucasian	304	441	Population based	NA	NA	NA	432 (71)	176 (29)	NA	NA	NA	564 (64)	318 (36)	8
Su	China	2005	Asian	84	97	Not mentioned	61	21	2	143 (85)	25 (15)	54	35	8	143 (74)	51 (26)	7
van Diemen	Netherlands	2006	Caucasian	183	1145	Population based	106	67	10	279 (76)	87 (24)	584	474	87	1642 (72)	648 (28)	7
Yoon	Korea	2006	Asian	102	159	Hospital based	21	54	27	96 (47)	108 (53)	48	74	37	170 (53)	148 (47)	8
Ito	Japan	2008	Asian	70	99	Hospital based	NA	NA	NA	78 (56)	62 (44)	NA	NA	NA	101 (51)	97 (49)	7
Mak	China	2009	Asian	202	195	Population based	35	97	70	167 (41)	237 (59)	36	90	69	162 (42)	228 (58)	7
Chappell	U.K.	2011	Caucasian	1017	912	Not mentioned	NA	NA	NA	1383 (68)	651 (32)	NA	NA	NA	1240 (68)	584 (32)	7
Yuan	China	2011	Asian	117	82	Hospital based	31	59	27	121 (52)	113 (48)	22	45	15	89 (54)	75 (46)	7
Guo	China	2012	Asian	326	208	Hospital based	74	167	85	315 (48)	337 (52)	39	104	65	182 (44)	234 (56)	8

* Score of NOS. Abbreviation: NA, not applicable.

**Table 1.3:** Characteristics of the studies of rs2241712 and COPD risk

Author	Country	Year	Ethnicity	Cases (*n*)	Controls (*n*)	Control origin	Cases					Controls					Score*
							GG	GA	AA	G allele (%)	A allele (%)	GG	GA	AA	G allele (%)	A allele (%)	
Celedon	U.S.A.	2004	Caucasian	304	441	Population based	NA	NA	NA	505 (83)	103 (17)	NA	NA	NA	732 (83)	150 (17)	8
Yoon	Korea	2006	Asian	102	159	Hospital based	30	51	21	111 (54)	93 (46)	41	75	43	157 (49)	161 (51)	8
Ito	Japan	2008	Asian	70	99	Hospital based	NA	NA	NA	78 (56)	62 (44)	NA	NA	NA	101 (51)	97 (49)	7
Guo	China	2012	Asian	331	207	Hospital based	81	158	92	320 (48)	342 (52)	63	100	44	226 (55)	188 (45)	8

* Score of NOS. Abbreviation: NA, not applicable.

**Table 1.4:** Characteristics of the studies of rs6957 and COPD risk

Author	Country	Year	Ethnicity	Cases (*n*)	Controls (*n*)	Control origin	Cases					Controls					Score*
							GG	GA	AA	G allele (%)	A allele (%)	GG	GA	AA	G allele (%)	A allele (%)	
Celedon	U.S.A.	2004	Caucasian	304	441	Population based	NA	NA	NA	103 (17)	505 (83)	NA	NA	NA	150 (17)	732 (83)	8
van Diemen	Netherlands	2006	Caucasian	184	1128	Population based	103	71	10	277 (75)	91 (25)	771	327	30	1869 (83)	387 (17)	7
Ito	Japan	2008	Asian	70	99	Hospital based	NA	NA	NA	108 (77)	32 (23)	NA	NA	NA	154 (78)	44 (22)	7
Chappell	U.K.	2011	Caucasian	1017	912	Not mentioned	NA	NA	NA	346 (17)	1688 (83)	NA	NA	NA	328 (18)	1496 (82)	7
Gong	China	2011	Asian	157	171	Hospital based	57	77	23	191 (61)	123 (39)	60	89	22	209 (61)	133 (39)	8

* Score of NOS. Abbreviation: NA, not applicable.

**Table 1.5:** Characteristics of the studies of rs2241718 and COPD risk

Author	Country	Year	Ethnicity	Cases (n)	Controls (n)	Control origin	Cases					Controls					Score*
							TT	TC	CC	T allele (%)	C allele (%)	TT	TC	CC	T allele (%)	C allele (%)	
Celedon	U.S.A.	2004	Caucasian	304	441	Population based	NA	NA	NA	103 (17)	505 (83)	NA	NA	NA	150 (17)	732 (83)	8
Ito	Japan	2008	Asian	70	99	Hospital based	NA	NA	NA	116 (83)	24 (17)	NA	NA	NA	170 (86)	28 (14)	7
Guo	China	2012	Asian	331	206	Hospital based	26	154	151	206 (31)	456 (69)	10	94	102	114 (28)	298 (72)	8

* Score of NOS. Abbreviation: NA, not applicable.

### Meta-analysis of the *TGF-β1* polymorphisms

**Figure 2:**
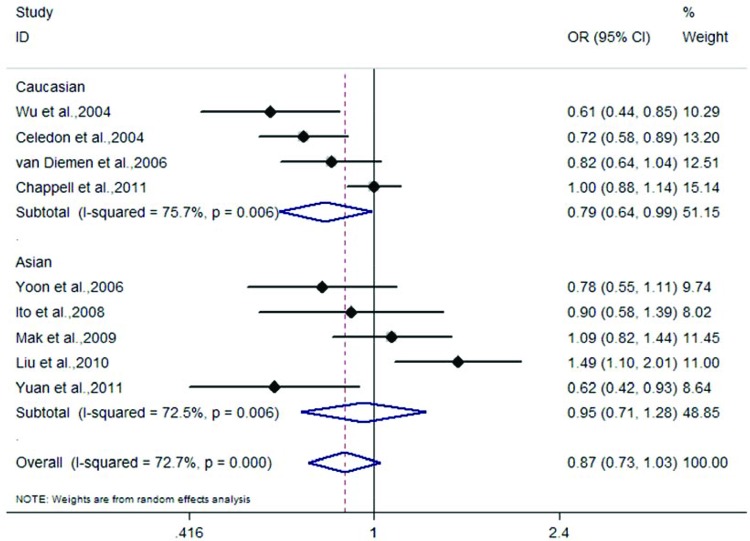
Meta-analysis of the association between rs1982073 and COPD risk in allelic comparison model (C allele compared with T allele)

Nine case–control association studies including 2451 cases and 3247 controls evaluated the *TGF-β1* rs1982073 polymorphism. By pooling these nine studies, meta-analysis showed no association between this polymorphism and COPD risk in all the subjects (Caucasians and Asians) in dominant (OR =0.81, 95% CI: 0.59–1.12, *P*=0.206), recessive (OR =0.94, 95% CI: 0.51–1.74, *P*=0.849), homozygote (OR =0.74, 95% CI: 0.40–1.37, *P*=0.342), and allelic comparison (OR =0.87, 95% CI: 0.73–1.03, *P*=0.109) models (Figure 2 and Table 2). However, in subgroup analysis according to ethnicity, we found that the C allele of rs1982073 was associated with decreased risk of COPD in Caucasians (OR =0.79, 95% CI: 0.64–0.99, *P*=0.038) (Figure 2 and Table 2). We did not find an association between this polymorphism and COPD risk in Asians in any of the genetic risk models (Figure 2 and Table 2).

**Table 2:** Meta-analysis of the *TGF-β1* gene polymorphisms and COPD risk

Variant	Dominant			Recessive			Homozygote			Allelic contrast		
	OR (95% CI)	*P*_z_	*P*_h_	OR (95% CI)	*P*_z_	*P*_h_	OR (95% CI)	*P*_z_	*P*_h_	OR (95% CI)	*P*_z_	*P*_h_
rs1982073												
All	0.81 (0.59–1.12)	0.206	0.025	0.94 (0.51–1.74)	0.849	<0.001	0.74 (0.40–1.37)	0.342	<0.001	0.87 (0.73–1.03)	0.109	<0.001
Caucasian	0.66 (0.50–0.86)	0.002	0.503	0.61 (0.26–1.46)	0.269	0.039	0.50 (0.20–1.22)	0.128	0.048	0.79 (0.64–0.99)	0.038	0.006
Asian	0.94 (0.60–1.47)	0.771	0.056	1.17 (0.53–2.58)	0.697	<0.001	0.92 (0.41–2.08)	0.842	0.001	0.95 (0.71–1.28)	0.741	0.006
rs1800469												
All	0.88 (0.66–1.18)	0.398	0.069	0.89 (0.72–1.11)	0.315	0.352	0.87 (0.66–1.14)	0.318	0.114	0.89 (0.77–1.02)	0.099	0.019
Caucasian	NA	NA	NA	NA	NA	NA	NA	0.192	NA	0.84 (0.68–1.05)	0.126	0.030
Asian	0.93 (0.64–1.35)	0.694	0.055	0.92 (0.73–1.17)	0.501	0.291	0.93 (0.69–1.26)	0.651	0.101	0.89 (0.77–1.02)	0.449	0.051
rs2241712												
All	1.15 (0.84–1.58)	0.385	0.163	1.03 (0.52–2.07)	0.927	0.053	1.08 (0.45–2.58)	0.860	0.041	1.03 (0.89–1.20)	0.666	0.123
Asian	1.15 (0.84–1.58)	0.385	0.163	1.03 (0.52–2.07)	0.927	0.053	1.08 (0.45–2.58)	0.860	0.041	1.05 (0.88–1.26)	0.589	0.059
rs6957												
All	1.30 (0.74–2.30)	0.368	0.039	1.45 (0.89–2.36)	0.134	0.227	1.52 (0.91–2.56)	0.111	0.111	1.14 (0.95–1.36)	0.160	0.084
Caucasian	NA	NA	NA	NA	NA	NA	NA	NA	NA	1.19 (0.92–1.54)	0.194	0.023
Asian	NA	NA	NA	NA	NA	NA	NA	NA	NA	1.02 (0.78–1.33)	0.893	0.937
rs2241718												
All	NA	NA	NA	NA	NA	NA	NA	NA	NA	0.95 (0.79–1.14)	0.571	0.428
Asian	NA	NA	NA	NA	NA	NA	NA	NA	NA	0.91 (0.71–1.16)	0.431	0.237

Abbreviations: NA, not applicable; *P*_h_, *P*-value for heterogeneity; *P*_z_, *P* value for overall effect.

Nine studies with a total of 2405 COPD patients and 3338 controls assessed the relationship of rs1800469 with COPD. Pooling the data from these studies provided no evidence that this polymorphism influenced COPD risk in dominant (OR =0.88, 95% CI: 0.66–1.18, *P*=0.398), recessive (OR =0.89, 95% CI: 0.72–1.11, *P*=0.315), homozygote (OR =0.87, 95% CI: 0.66–1.14, *P*=0.318), and allelic comparison (OR =0.89, 95% CI: 0.77–1.02, *P*=0.449) models (Table 2). Similarly, no association was found between rs2241712, rs6957, rs2241718, and COPD risk (Table 2).

### Heterogeneity, sensitivity analysis, and publication bias

Between-study heterogeneity was identified in the pooled analyses of rs1982073, rs2241712, rs1800469, and rs6957, but not in those for rs2241718 (Table 2). The DerSimonian and Laird random effects meta-analysis model was used to calculate pooled ORs in the presence of heterogeneity. Sensitivity analyses were performed applying the one-study remove approach to assess the impact of each study on the combined effect. We reran analyses after excluding each study, and results were similar to the initial analyses (results not shown). Publication bias was not found using the Begg’s test (Table 3).

**Table 3:** Evaluation of publication bias using Begg’s test

Genetic risk model	Polymorphism				
	rs1982073	rs1800469	rs2241712	rs6957	rs2241718
Dominant	0.452	0.707	1.000	1.000	NA
Recessive	0.060	1.000	1.000	1.000	NA
Homozygote	0.260	1.000	1.000	1.000	NA
Allelic contrast	1.000	0.917	0.308	0.806	0.296

## Discussion

We carried out a meta-analysis of published data on the relation of the *TGF-β1* SNPs rs1982073, rs1800469, rs2241712, rs6957, and rs2241718 with COPD in populations of different origins. The main findings of our study are the following: (i) the C allele of rs1982073 is associated with decreased risk of COPD in Caucasians, whereas there is no association between rs1982073 and COPD risk in Asians; (ii) the *TGF-β1* polymorphisms rs1800469, rs2241712, rs6957, and rs2241718 are not associated with COPD risk.

The results of rs1982073 in the present study were not consistent with two prior meta-analyses which showed that the C allele of rs1982073 was associated with decreased risk of COPD in both Caucasians and Asians [28]. Our meta-analysis did not suggest an association of rs1982073 with COPD in Asians. For evaluating rs1982073 in Asians, a study whose genotype distribution in the control group showing deviation from HWE was inappropriately included in the meta-analysis by Zhang et al., which may bias the pooled effect size in Asians [25]. We did not include that study in our meta-analysis. Besides this discrepancy, we included a recently published Asian study in our analyses [15]. Because of strict selection of eligible studies, our results were more reliable. In 2009, the meta-analysis by Smolonska et al. [29] evaluated the *TGF-β1* polymorphisms and found that there was an association of rs2241712 and rs6957 with COPD risk using a small number of subjects. However, these results were not confirmed by our meta-analysis including more eligible studies. For evaluating rs1982073, Smolonska et al. [29] combined data from five studies including subjects of different ethnic groups, but did not conduct subgroup analysis according to ethnicity in Caucasians and Asians, respectively. Our meta-analysis provided more valuable information on the relationship of rs1982073 with COPD because we performed ethnicity specific analysis. Compared with previous meta-analyses, there were some advantages in our study. First, we carefully evaluated the quality of each study. Second, we checked for HWE in control subjects and excluded studies showing deviation from HWE. Third, we evaluated the relation of five common *TGF-β1* polymorphisms with COPD; the number of SNPs was the largest amongst all meta-analyses on this topic.

TGF-β1 is a multifunctional cytokine which regulates cell proliferation and differentiation, immune responses, and production of collagen and other extracellular matrix proteins. Animal studies have shown that mice with abnormalities in the activation and signaling of TGF-β1 develop pulmonary emphysema through increased expression of the extracellular macrophage metalloproteinase Mmp12 in the lung [30]. The latent TGF-β1-binding proteins (LTBPs) are responsible for the export and deposition of TGF-β1 in the extracellular matrix. Mutant mice generated by a gene trap integration into the mouse *LTBP-4* gene developed severe pulmonary emphysema owing to a reduced deposition of TGF-β1 in the extracellular space [31]. Moreover, in a mouse model of COPD, activation of the TGF-β1 signaling pathway resulted in a dramatic recovery of the pulmonary emphysema phenotype [32]. These studies strongly suggested that TGF-β1 was protective against COPD development. The *TGF-β1* polymorphism rs1982073 is a C→T substitution, resulting in a proline to leucine substitution at codon 10 [33]. It was reported that the C allele of rs1982073 was associated with higher *TGF-β1* mRNA and protein levels [34,35]. Thus, the negative association of the C allele with COPD risk may partly be due to its correlation with higher TGF-β1 production.

The present study has potential limitations. First, we did not assess the relation of the *TGF-β1* polymorphisms with lung function in COPD patients and disease severity, because there were not enough published studies in this field. The study by van Diemen et al. [36], found that the *TGF-β1* polymorphisms were associated with forced expiratory volume in 1 s (FEV1)/inspiratory vital capacity (IVC) in patients with COPD, suggesting that the severity of airway obstruction was genetically influenced by the *TGF-β1* polymorphisms. Future studies are needed to validate their findings in different populations. Second, gene–gene interaction was not investigated in this meta-analysis because most included studies did not address this issue. Third, we only included common *TGF-β1* SNPs assessed in more or equal than three studies. Other *TGF-β1* SNPs, such as rs1800471 and rs1800468, were not evaluated in this meta-analysis due to the limited availability of published results. Fourth, we did not conduct stratified analysis to adjust for smoking due to limited availability of published data.

In summary, we found that the C allele of rs1982073 was associated with decreased risk of COPD in Caucasians, but not in Asians. There was no association of rs1800469, rs2241712, rs6957, and rs2241718 with COPD risk.

## Abbreviations

CI: confidence interval
COPD: chronic obstructive pulmonary disease
HWE: Hardy–Weinberg equilibrium
LTBP: latent TGF-β1-binding protein
OR: odds ratio
SNP: single nucleotide polymorphism
TGF-β1: transforming growth factor-β1
